# Dentofacial abnormalities among adolescents: A study on the prevalence and severity

**DOI:** 10.4317/jced.52246

**Published:** 2015-04-01

**Authors:** Rekha P. Shenoy, Ganesh Shenoy-Panchmal

**Affiliations:** 1 Reader, Department of Public Health Dentistry, Yenepoya Dental College, Mangalore, India; 2 Professor and Head, Department of Public Health Dentistry, Yenepoya Dental College, Mangalore, India

## Abstract

**Background:**

The objectives of this investigation were to assess prevalence and severity of dentofacial abnormalities and orthodontic treatment need among adolescents in Mangalore taluk.

**Material and Methods:**

A cross-sectional study was conducted among 1340 children from randomly selected high schools. A proforma was used to record demographic data and components of the Dental Aesthetic Index [DAI] for each subject. The Chi squared test was used for analysis with p-value of < 0.05 considered statistically significant.

**Results:**

Dentofacial abnormalities (DAI scores ≥ 26) were recorded in 38.5% subjects. Mean DAI score of the study population was 24.59 ± 6.09. Female subjects presented with higher prevalence and higher mean DAI scores than their male counterparts (p > 0.05). Assessment of severity of malocclusion between age groups revealed no differences (p > 0.05). Orthodontic treatment was highly desirable in 11% and mandatory in 5.2% subjects.

**Conclusions:**

A high prevalence of dentofacial abnormalities was found among adolescents in Mangalore taluk pointing towards a need for designing effective programs for early diagnosis and treatment of this condition, especially among adolescents.

** Key words:**Adolescents, Dental Aesthetic Index [DAI], dentofacial abnormalities, malocclusion, orthodontic treatment need, prevalence, severity.

## Introduction

Societal forces define norms for an acceptable physical appearance and equate good dental appearance with success in life (1,2). An increased concern for dental appearance has been observed during adolescence and early adulthood (2,3). Malocclusion can be described as an irregularity of the teeth or a poor relationship of the dental arches beyond the range of what is accepted as normal (1,2). It is the third most prevalent oral pathology and, therefore, ranks third among dental public health priorities (1). Malocclusion can impact quality-of-life causing psychosocial limitations (awkwardness in the social context or reduced career opportunities) and functional disturbances (affecting mastication, swallowing and speech; increasing susceptibility to trauma; and increasing prevalence of dental caries, periodontal disease and temporomandibular joint disorders) (1-3).

Among the various indices / methods used to evaluate malocclusion, the WHO-recommended Dental Aesthetic Index (DAI), developed in 1986, has proven to be a simple, reproducible and rapidly applied cross-cultural index that links clinical and aest-hetic components mathematically to produce a single score (1-5). This index can be used for different populations without modification (1).

Documentation of the prevalence and severity of a condition is crucial for formulation of health policies and treatment programs. Mangalore is one among the five taluks/subdivisions of Dakshina Kannada District of Karnataka State, India and a reputed centre for medical education and health care. A search of scientific literature revealed the existence of a lacuna in available data on the prevalence of dentofacial abnormalities among adolescents in this region of India. Therefore, this study was conducted to assess the prevalence and severity of dentofacial abnormalities and orthodontic treatment need among adolescents in Mangalore taluk.

## Material and Methods

A cross-sectional study was conducted for a fourteen-month period from October 2012 to November 2013. Schools in Mangalore taluk are divided into two administrative blocks (City Range and Rural), each governed through a Block Educational Officer (BEO). High schools numbered 70 in Mangalore City Range and 121 in Mangalore Rural.

-Sample size: Based on available data, sample size was determined to be 1340 (confidence level – 95%, power of the test – 90%). As the number of children in each school was unknown, it was decided that 670 children each would be examined in each block from randomly selected schools. During the sampling procedure, equal representation was given to subjects from both urban and rural areas, and to those enrolled in public and private schools, thus ensuring coverage of all population subgroups likely to have differing levels of oral disease or treatment needs. Permission to carry out the study was obtained from the BEOs, school authorities and the Institutional Ethics Committee.

-Data collection: A proforma was used to record demographic data and the DAI criteria of each subject. In the selected schools, all students who fulfilled the following inclusion criteria were examined till the required sample was obtained: (a) male and female children present on the day of survey, and (b) consenting to participate in survey. Those who had undergone or were undergoing orthodontic treatment were excluded (1,2). Informed consent was obtained from all participants.

Examinations were conducted under adequate natural light using plane mouth mirrors and WHO Periodontal Probes with subjects seated on chairs in classrooms or school corridors. Instruments were autoclaved before use and a maximum of 25 subjects were examined per day with the help of a recorder. The investigator underwent training and calibration prior to data collection and the kappa value for intra-examiner reliability was 0.9. To reduce bias, duplicate examinations were conducted on 70 subjects during the study and intra-examiner variability was found to be less than 1%.

-Statistical analysis: Data were analyzed using the SPSS Version 17.0 program for Windows. Differences in proportions between the different age groups and genders were compared using the Chi squared test and a *p*-value of < 0.05 was considered statistically significant.

## Results

Total number of study subjects, who consented to participate and were examined, numbered 1340 ( Table 1) and belonged to ten schools. They ranged in age from 11 - 18 years (mean age 13.91 ± 1.17 years). Exclusions numbered twenty three. As the number of subjects in the 11- and 18-year age groups was insignificant, for analysis, the five 11-year-olds were included in the 12-year age group while the only 18-year-old was added to the 17-year age group.

**Table 1:**
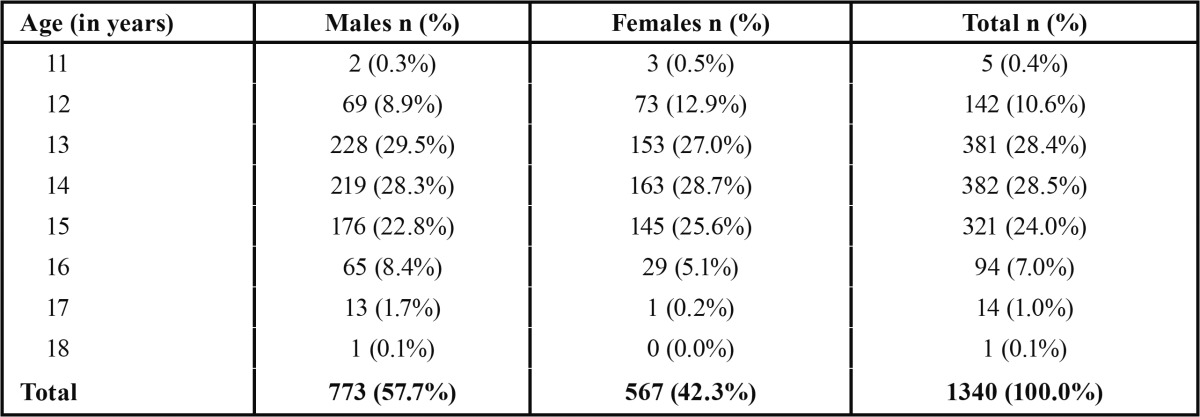
Age and gender distribution of the study subjects.

 Table 2 shows distribution of components of the DAI among study subjects. Crowding, spacing, diastema, maxillary irregularity, increased mandibular overjet and openbite were most prevalent in the 17-year age group. Also, female subjects presented with higher values for crowding, diastema, increased maxillary overjet, openbite and molar relation anomalies (*p* > 0.05). Analysis of components of the DAI for different age groups revealed insignificant differences (*p* > 0.05).

**Table 2:**
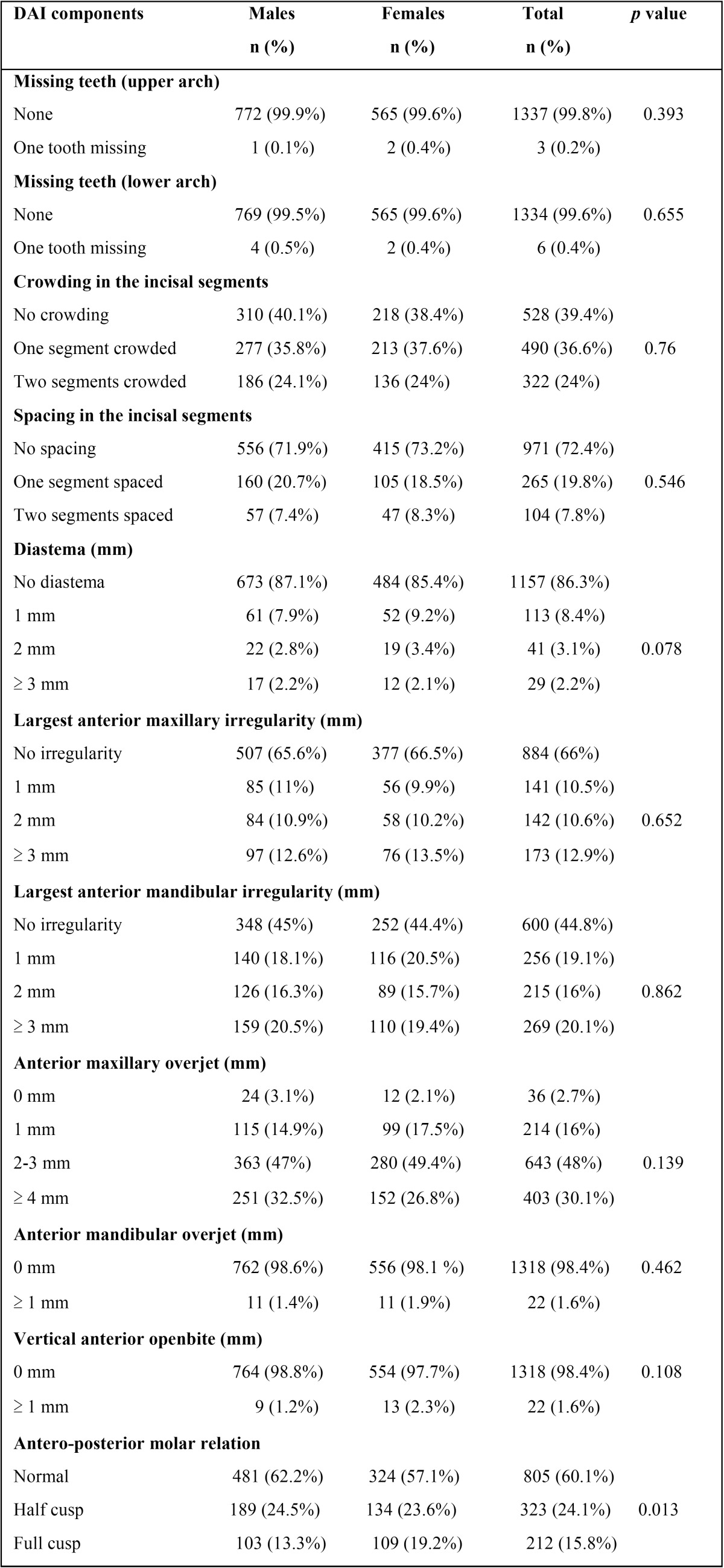
Distribution of the DAI components among study subjects.

Dentofacial abnormalities (scores ≥ 26) were found in 38.5% subjects (38.2% males and 39% females). Distribution of subjects according to the severity of malocclusion was as follows: definite malocclusion (scores 26 – 30) was found in 22.2% subjects (22.9% males and 21.3% females), severe malocclusion (scores 31 – 35) in 11% subjects (10.2% males and 12.2% females), and very severe or handicapping malocclusion (scores ≥ 36) in 5.2% subjects (5% males and 5.5% females). No significant differences existed between genders for the different grades of severity (*p* = 0.653). Assessment of severity of malocclusion across different age groups revealed no differences (*p* = 0.583). Orthodontic treatment need of the population was as follows: 61.5% subjects required little or no orthodontic treatment; it was an elective option in 22.2% subjects; highly desirable in the 11% presenting with severe malocclusion and mandatory in the 5.2% with very severe or handicapping malocclusion. Mean DAI score for the study population was 24.59 ± 6.09. Mean DAI scores for male (24.51 ± 6.02) and female (24.69 ± 6.19) subjects showed insignificant differences (*p* = 0.586). Evaluation between age groups also revealed no differences (*p* = 0.759).

## Discussion

The number of subjects examined and their age range were higher than studies conducted in India and abroad (1-4,6). However, male predominance seen in other studies (1,2) was reflected here.

-Missing anterior teeth: Proportion of subjects with missing anterior teeth was minimal (0.7%) in comparison to investigations by Tak *et al.* (10.5%) (1), Shivakumar *et al.* (11%) (2), Marques *et al.* (22.3%) (4) and Gabris *et al.* (11.2%) (6). Differences between genders and age groups were found to be insignificant (1). However, Shivakumar *et al.* (2) found significant differences while comparing between genders.

-Crowding in the incisal segments: In contrast to the maxilla, the mandibular arch has less space available (the “incisor liability”) for the four incisors to align perfectly. Crowding was observed in almost two-third of the subjects (60.6%) which was higher than reported by Tak *et al.* (40.2%) in Udaipur (1), Shivakumar *et al.* (38.2%) in Davangere (2), Marques *et al.* (47.3%) in Brazil (4) and Gabris *et al.* (14.3%) in Hungary (6). In contrast to the findings of Tak *et al.* (1), prevalence was higher among female subjects and the older age groups, although the differences were insignificant (*p* > 0.05) (2). One segment crowding predominated over two segments’ crowding (1) while Gabris *et al.* (6) found an equal occurrence of both types. The study population had more crowding than spacing (1).

-Spacing in the incisal segments: Arch continuity (proximal contact between all teeth in each dental arch) facilitates optimal dental function (2). Observed among 27.6% subjects, spacing was higher than reported by Gabris *et al.* (17%) (6) but in accordance with the findings of Tak *et al.* (27.1%) (1) and Shivakumar *et al.* (26.5%) (2). Subjects with 1 segment spacing were more than twice those with 2 segments’ spacing (6). Age (1) and gender (1,2) were not found to influence occurrence of spacing.

-Diastema: It was recorded in 13.7% adolescents which was more than reported by Gabris *et al.* (7.8%) (6). Other studies (1,2) have reported a higher prevalence of between 15.3% and 18.3%. Prevalence was higher in females (2), in contrast to another study (1). Age, however, was not an influencing factor (1). During the mixed dentition period, diastema is a frequently occurring variation in the development pattern. Persistence after eruption of permanent lateral incisors and the unaesthetic appearance of spaced upper incisors has been termed the “Ugly Duckling Stage”.

Incisor crowding and midline diastema may have the greatest negative impact on perceived beauty and thereby, self-esteem (7).

-Largest anterior maxillary irregularity: Prevalence was lower (34%) than reported by Tak *et al.* (45.7%) (1) and Gabris *et al.* (56.7%) (6), while a study in Davangere found 25.6% prevalence (2). These differences could be due to genetic and environmental factors (2). Occurrence of irregularity declined from 17 years to 13 years (*p*>0.05), a reverse of the phenomenon reported by Tak *et al.* (1). Prevalence was almost equal between genders in this study (2) while Tak *et al.* (1) found a significantly higher proportion of affected males.

-Largest anterior mandibular irregularity: Mandibular irregularities affected 55.2% subjects, higher than other reports (1,2). Analogous findings were, however, reported by Gabris *et al.* (41.8%) (6). The prevalence was uninfluenced by gender (1,2) or age (1). These contrasting results could be due to genetic and racial composition of the study groups, and environmental influences (2). While Tak *et al.* (1) reported that irregularities occurred more frequently in the upper arch, our findings showed a predominance in the mandible.

-Anterior maxillary overjet: ≥ 4 mm overjet was found among 30.1% subjects. Tak *et al.* (1) found 12.7% subjects with ≥ 4 mm overjet while Marques *et al.* (4) reported 21.8% prevalence of > 3 mm overjet. There were no differences between genders (1,2) or age groups (1) for this parameter. Edge to edge bite was recorded in 2.7% cases, higher than found by Tak *et al.* (1.4%) (1). Teeth with increased overjets are prone to traumatic injuries (8) and difficulty in cleansing (9).

-Anterior mandibular overjet: It was evident in 1.6% subjects, similar to other reports (1,6). Shivakumar *et al.* (2) reported 0.3% subjects with 1-2 mm mandibular overjet. Findings were insignificant for gender (1,2) and age (1).

-Vertical anterior openbite: Present in 1.6% examinees, it was lower than reported by Tak *et al.* (2.5%) (1), Shivakumar *et al.* (2.1%) (2) and Gabris *et al.* (10.8%) (6). Occurrence of openbite declined from 17 to 15 years. Again, age (1) and gender (1,2) were inconsequential factors.

Occurrence of spacing, diastema, mandibular overjet and openbite may be genetically determined, attributable to dento-alveolar discrepancies or to deleterious oral habits (1,2).

-Antero-posterior molar relation: Deviations (half and full cusp), although similar to the observations of Gabris *et al.* (6), were higher than other reports (1,2). Half cusp deviation was the more frequently obtained finding (1,2,6). Significant differences existed between genders (*p* = 0.013), in contrast to other studies (1,2). However, no differences were found between the age groups (1).

Prevalence of dentofacial abnormalities (38.5%) was lower than reported in Brazil and Hungary [Marques *et al.* (4) - 77%, Gabris *et al.* (6) - 70.4%, respectively]. This study population presented with greater treatment need than observed in other Indian studies in Udaipur and Davangere [Tak *et al.* (1) – 33.3%, Shivakumar *et al.* (2) – 19.9%, respectively]. These dissimilar results may be attributed to ethnic, physical and cultural differences among the populations studied. Assessment of the severity of malocclusion between genders (2) and age groups (2) revealed no significant differences, in contrast to Tak *et al.* (1).

Mean DAI score for this study population was higher than reported by Tak *et al.* (1). Mean DAI scores for males and females were also higher than another report (1). Mean DAI score was higher among females when compared to their male counterparts; while Tak *et al.* (1) found higher scores among males, Marques *et al.* (4) found no significant differences between genders. Mean DAI scores showed no differences among age groups whereas Tak *et al.* reported reduction in scores with age and reasoned that temporary malocclusions self-correct with age and most children outgrow deforming habits returning dental relationships to normal (1).

To conclude, prevalence of dentofacial abnormalities among adolescents of Mangalore taluk was found to be 38.5%. Implementation of programs for the early diagnosis and treatment of this condition will go a long way in intercepting its progression and in improving quality of life among affected individuals.
